# Regulation of Wnt/β-catenin signaling by Marek’s disease virus *in vitro* and *in vivo*

**DOI:** 10.3389/fmicb.2024.1388862

**Published:** 2024-04-03

**Authors:** Haiyin Xu, Xihao Xu, Huifeng He, Hongxia Shao, Yongxiu Yao, Aijian Qin, Kun Qian

**Affiliations:** ^1^Ministry of Education Key Laboratory for Avian Preventive Medicine, Yangzhou University, Yangzhou, China; ^2^Jiangsu Key Laboratory of Preventive Veterinary Medicine, Yangzhou University, Yangzhou, China; ^3^Jiangsu Co-innovation Center for Prevention and Control of Important Animal Infectious Diseases and Zoonoses, Yangzhou University, Yangzhou, Jiangsu, China; ^4^The Pirbright Institute & UK-China Centre of Excellence for Research on Avian Diseases, Surrey, United Kingdom

**Keywords:** Marek’s disease virus, Wnt/β-catenin signaling pathway, RB1B, CVI988, Meq

## Abstract

Marek’s disease virus (MDV) infection causes immunosuppression in the host, ultimately inducing tumor formation and causing significant economic losses to the poultry industry. While the abnormal activation of the Wnt/β-catenin signaling pathway is closely associated with the occurrence and development of tumors. However, the relationship between MDV and the Wnt/β-catenin pathway remains unclear. In this study, we found that the MDV RB1B strain, but not the MDV vaccine strain CVI988, activated the Wnt/β-catenin signaling pathway by increasing the phosphorylation level of GSK-3β in chicken embryo fibroblast (CEF). *In vivo* infection experiments in SPF chickens also confirmed that the RB1B strain activated the Wnt/β-catenin signaling pathway, while the CVI988 strain did not lead to its activation. Moreover, unlike the Meq protein encoded by the CVI988 strain, the Meq protein encoded by the RB1B strain specifically activated the Wnt/β-catenin signaling pathway in CEF cells. The findings from these studies extend our understanding of the regulation of Wnt/β-catenin signaling by MDV, which make a new contribution to understanding the virus–host interactions of MDV.

## Introduction

Marek’s disease virus (MDV) belongs to the family α herpesviridae and subgroup B herpesvirus The virus infection causes proliferation of T lymphocytes and tumor formation, leading to immunosuppression in chicken flocks, reduced feed conversion rate, decreased egg production, and weight loss, resulting in significant economic losses to the poultry industry (Zhou et al., 2022). In clinical practice, although vaccines such as CVI988, 814, and HVT were used to prevent the economic losses caused by virulent MDV-induced tumors and immunosuppression, they could not eliminate the replication of virulent strains in chickens. Meanwhile, the virulence of MDV was continuously evolving. Therefore, there were sporadic reports of MDV infections in chicken flocks that had been vaccinated against MDV (Baigent et al., 2011, 2016). Thus, the progress in understanding the mechanisms underlying MDV pathogenesis and tumorigenesis was particularly important for the development of novel prevention and control measures as well as vaccines against MDV.

Recently, researchers had made some progress in the study of immunosuppression and tumorigenesis of MDV. Gao et al. (2019) discovered that MDV-encoded VP23 protein interacted with IRF7, inhibiting its phosphorylation and nuclear translocation, thereby reducing IFN-β production and promoting viral replication. Research by Li et al. (2019) showed that the Meq protein of MDV inhibited the expression of IFN-β by recruiting IRF7 and TBK1. Moreover, Meq also suppressed the CD8+ T cell response, inducing lymphoma formation (Liu and Kung, 2000; Burgess et al., 2004; Lupiani et al., 2004). Li et al. (2018) discovered that MDV infection could activate the PI3K/Akt signaling pathway, inhibiting cell apoptosis and promoting viral replication. Recent findings also indicated that miR-M11 was a non-essential gene for MDV replication, and its deletion could enhance the *in vitro* replication ability of MDV (Wang et al., 2023). Additionally, gga-miR-219b, gga-miR-130b-3p, and gga-miR-140b-3p played inhibitory roles in the formation of MD tumors, whereas the target gene BCL11B of gga-miR-219b and the Meq promoted tumor formation (Zhao et al., 2017). The recent findings reported above provide a basis for explaining the pathogenic mechanism of MDV.

The Wnt/β-catenin signaling pathway plays a pivotal role in cell development and differentiation and is implicated in developmental disorders and cancer (Anastas and Moon, 2013; Duchartre et al., 2016). β-catenin, as the central regulatory protein in the Wnt/β-catenin signaling pathway, is present in the cytoplasm. In the absence of Wnt ligands, it undergoes phosphorylation by GSK-3β and is degraded through the proteasome pathway. Therefore, β-catenin cannot translocate into the nucleus to bind with T-cell factor/lymphoid enhancer factor (TCF/LEF) and activate the signaling pathway (Nusse and Clevers, 2017). When Wnt ligands are present, β-catenin accumulates within the cell and undergoes nuclear translocation. β-catenin enters the cell nucleus and binds with the TCF/LEF transcription factor family, thereby activating the Wnt pathway and inducing the expression of target genes (Taciak et al., 2018). Research has confirmed that many viruses interact with the Wnt/β-catenin signaling pathway, altering it in various ways to achieve their objectives. For instance, the negative regulatory factor of HIV interacted with β-catenin, inhibiting Wnt/β-catenin signal transduction (Kumar et al., 2008). Tran et al. (2020) demonstrated that the Hepatitis B Virus (HBV) precursor cellular protein p22 could activate Wnt signaling in a cancer environment. In livestock and poultry diseases, it was found that the ability of β-catenin to maintain the growth potential of non-neuronal cells stimulated productive infection by Bovine Herpesvirus-1 (BoHV-1) (Zhu et al., 2017). Infection with Porcine Circovirus-Like Virus P1 was associated with the downregulation of the Wnt/β-catenin signaling pathway both *in vivo* and *in vitro* (Zhu et al., 2018). Previous reports from our laboratory had found that the activation of the classical Wnt/β-catenin signaling pathway was beneficial for the replication of ALV-J (Qiao et al., 2021). However, the effect of MDV infection on the Wnt/β-catenin signaling pathway is still unknown.

This study is the first time to investigate the effects of different MDV strains and the encoded Meq proteins on the Wnt/β-catenin signaling pathway both *in vitro* and *in vivo*, which providing a basis for further elucidation of the pathogenic mechanisms of MDV.

## Materials and methods

### Virus, cells and reagent

The RB1B and CVI988 strain of MDV were cultivated and preserved in our laboratory. Chicken embryo fibroblasts (CEF) were prepared using 9-day old SPF chicken embryos purchased from Zhejiang Lihua Co., Ltd., Zhejiang, China. Trypsin, DMEM, fetal bovine serum (FBS), Medium 199, Opti-MEM were all purchased from GIBCO (Shanghai, China). TransIT-X2 transfection reagent was purchased from Mirus (Dalian, China). Protease inhibitors, NP-40 were purchased from Beyotime (Shanghai, China). DMSO, iCRT14 and GAPGH antibody were purchased from SIGMA (Shanghai, China). TRIzol was purchased from Invitrogen (Shanghai, China). GSK-3β and p-GSK-3β (S9) monoclonal antibodies were purchased from Cell Signaling Technology (Shanghai, China). Avian β-catenin rabbit polyclonal antibody was prepared by Gene Script Bioscience and Technology Company (Nanjing, China). DNA/RNA extraction kit, reverse transcription kit, fluorescent quantitative PCR dye, and dual-luciferase reporter assay kit were all purchased from Vazyme (Nanjing, China). Wnt pathway transcription factor TCF/LEF reporter plasmids TOP flash and FOP flash were purchased from Merck (Shanghai, China).

The preparation of CEF was referred to previously report (Qiao et al., 2021). Nine-day-old chicken embryos with well-developed blood vessels and regular air chambers were selected. Using sterilized forceps, the embryos were removed and placed in PBS. The heads, limbs, and internal organs of the embryos were removed, leaving only the trunk, which was then placed in fresh PBS and gently rinsed to remove blood as much as possible. The clean tissues were minced in a petri dish, and then digested thoroughly in a ratio of 5 mL PBS to 0.05% trypsin per embryo, with the digestion process repeated 3–4 times. After digestion, the mixture was allowed to settle for 40 s, and the supernatant was collected and added to M199 medium containing 5% serum to terminate digestion. This digestion process was repeated 2–3 times until the supernatant became turbid and muscle tissue visibly decreased, indicating the completion of digestion. The cells after digestion were preliminarily filtered using a gauze funnel, then centrifuged at 800 rpm for 10 min to discard the supernatant. The cells were resuspended in M199 containing 5% serum medium and filtered through a 40 μm cell strainer. After cell counting, the cells were seeded into cell culture flasks and incubated in a 5% CO_2_, 37°C incubator.

### *In vivo* experiment

A total of 27 SPF chickens (white leghorn 1-day-old) were obtained from Boehringer Ingelheim (Jiangsu, China) and randomly divided into three groups. One group was as control group, one group was injected by MDV RB1B strain, and the other one was injected by MDV CVI988 strain. One-day-old chickens were infected with MDV RB1B and CVI988 strain intraperitoneally at a dose of 2000 PFU/chicken. The control group of chickens simulated infection with cell culture medium. Bursa of Fabricius, brain, spleen, liver and thymus were collected on 1, 4, and 7 days post infection (d.p.i.) to extract RNA for real-time PCR. The same tissue sample was taken from 3 chickens in each group.

### Real-time PCR assay

As previously reported (Qiao et al., 2021), the expression level of viral genes and target genes was determined by 7,500 Real-Time PCR System (ABI, United States). The sequences of all primers are listed in Table 1.

**Table 1:** Real-time PCR primer sequences.

Target of gene	Primers	Product length (bp)
18S	F5’- TCAGATACCGTCGTAGTTCC-3′ R5’- TTCCGTCAATTCCTTTAAGTT-3′	154
gB	F5’- ACCCCATTCGGTGGCTTTTC -3′ R5’- GCGTCCAGTTGTCTGAGG-3′	122
Meq	F5’-GTCCCCCCTCGATCTTTCTC-3′ R5’-CGTCTGCTTCCTGCGTCTTC-3′	184
β-catenin	F5’-ACAGCAAGGAACATGGCAAC-3′ R5’-CCACTCAAAGAGGGAGCAGT-3’	155
LEF1	F5’-CTTCAAGGACGAAGGGGACC-3′ R5’-GTTGACCAGCGAGGACTTGA-3’	103
TCF4	F5’-AAATCCCCCATCCGCTAGGA-3′ R5’-AGCCGACGTCACTCTGGGAA-3’	210
C-Myc	F5’-CAGAGGAGCACTGTAAGCCC-3′ R5’-AGCAGCGTAGTTGTGTTGGT-3’	83
Cyline D1	F5’-GCACAGCAGCACAACGTATC-3′ R5’-ATCTCGCACATCAGTGGGTG-3’	84

Total RNA from cells and tissues was extracted using the rapid RNA extraction kit (Vazyme, Nanjing, China), and 1 μg of RNA was reverse-transcribed into cDNA according to the manufacturer’s instructions using the HiScript III RT SuperMix for qPCR kit (Vazyme, Nanjing, China).

Real-time fluorescent quantitative PCR was used to detect the expression level of viral genes and target genes. The diluted cDNA (1 μL), 400 nM primers and 10 μL SYBR Green Master Mix were used for real-time PCR, and the reaction volume was 20 μL. The amplification conditions are as follows: 95°C for 30s, then 40 cycles, 95°C for 5 s, and 60°C for 34 s. After 40 cycles, a dissociation curve was generated to analyze each PCR product. The 2^-ΔΔCT^ method was used to analyze relative gene expression data with chicken 18S as an internal reference gene.

### Dual-luciferase reporter assay

As previously reported (Qiao et al., 2021), the luciferase Top flash plasmid with TCF-4 binding sites was transfected into CEF cells. At 12 h post-transfection, cells were either infected with the RB1B strain, the CVI988 strain, or transfected with the Meq plasmid. Samples were collected at different time points post-transfection. The transfection protocol involved using Mirus X2 reagent in a 24-well plate, with 500 ng of the target plasmid, 500 ng of Top flash/Fop flash, and 25 ng of pLR-TK co-transfected. Luciferase activity was monitored using a dual-luciferase reporter assay system according to the manufacturer’s protocol, and data were normalized based on Renilla luciferase activity.

### Western blot analysis

As previously reported (Qiao et al., 2021), the cells were lysed in RIPA buffer, and protein concentration was determined using the BCA Protein Assay Kit. Proteins (30 μg) were denatured by 100°C heating for 5 min. The denatured proteins (30 μg) were separated by electrophoresis under reducing conditions in a 12% SDS-PAGE. Proteins from the gel were transferred to a nitrocellulose membrane. The membrane was blocked with 5% non-fat milk in 0.1% Tween 20 at room temperature for 1 h and incubated overnight at 4°C with the appropriate primary antibody. The blots were washed three times with PBST, incubated with the corresponding enzyme-linked secondary antibody at 37°C for 45 min, and washed three times with PBST. Finally, the enhanced chemiluminescence (ECL) detection system (Pierce, Rockford, IL, United States) was used for imaging.

### iCRT14 treatment

According to the previous report (Qiao et al., 2021), the CEF cells were treated with iCRT14 (10 μM) following 12 h post-transfection of the Meq expression plasmid. The cell samples were collected at 24 h post-transfection for real-time PCR and dual-luciferase reporter assay.

### Statistical analysis of data

GraphPad Prism software (version 8.0) was used for graphing and analysis of data differences. Results in the figures represent the mean and standard error of three independent experiments. Differences between samples were analyzed using the Student’s *t-*Test. The *p*-value <0.05 indicates statistically significant difference.

## Results

### Effect of different strain of MDV infection on Wnt/β-catenin signaling pathway in CEF

To explore the effect of different MDV infection on the Wnt/β-catenin signaling pathway in CEF cells, the impacts of MDV RB1B and CVI988 strains on the pathway were studied. Dual-luciferase reporter assay was used to detect β-catenin/TCF transcriptional activity, and real-time PCR was performed to assess the expression of genes related to the Wnt/β-catenin signaling pathway.

**Figure 1:**
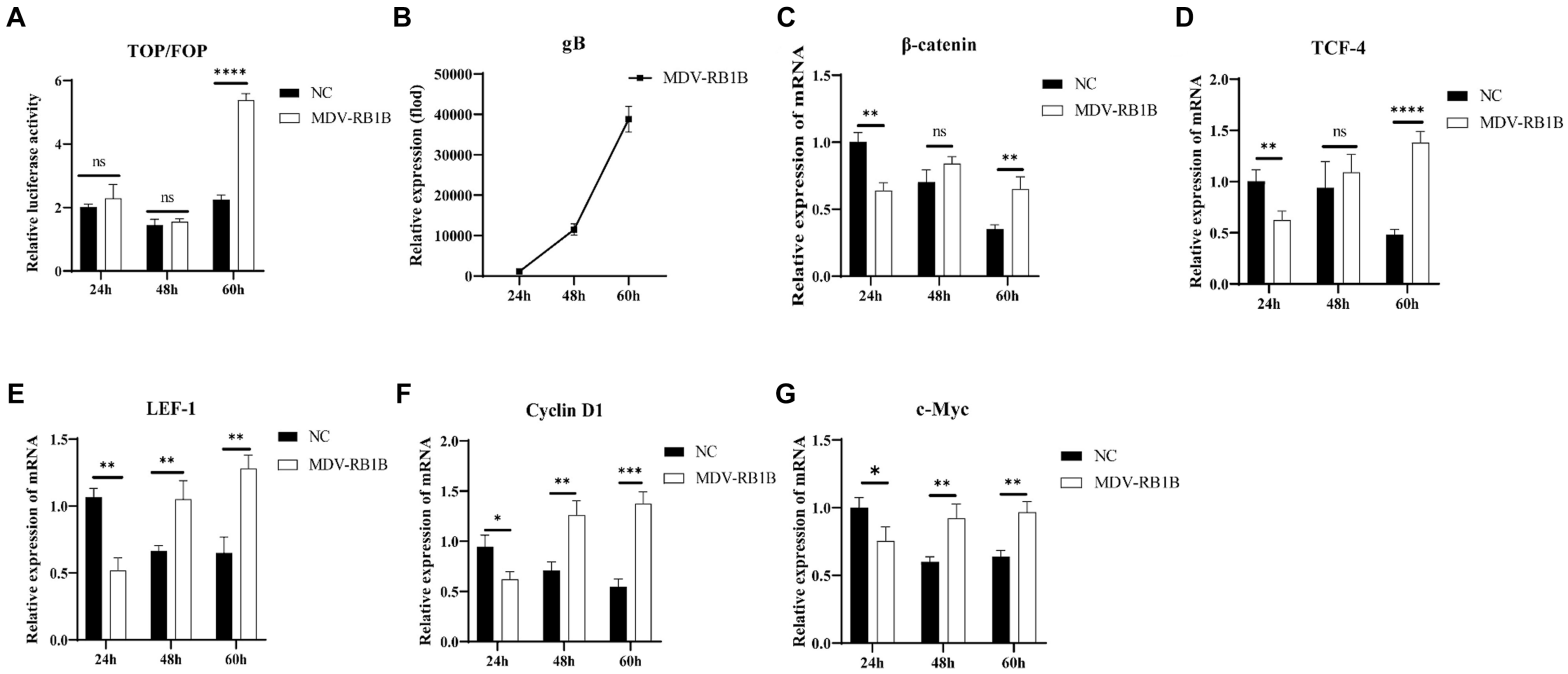
MDV RB1B activates the Wnt/β-catenin Signaling Pathway in CEF. Relative activity of TOP/FOP luciferase in CEF cells at 24, 48, and 60 h post-inoculation with MDV-RB1B strain **(A)**. Expression of MDV and Wnt/β-catenin signaling pathway-related genes, gB, β-catenin, LEF-1, TCF-4, Cyclin D1 and cMyc, in CEF cells at 24, 48, and 60 h post-inoculation with MDV-RB1B strain detected by real-time RT-PCR **(B–G)**. Data were expressed as mean ± SD from three independent experiments and analyzed by Student’s *t*-tests. And each experiment was repeated three times (^*^p < 0.05, ^**^p < 0.01, ^***^p < 0.001).

**Figure 2:**
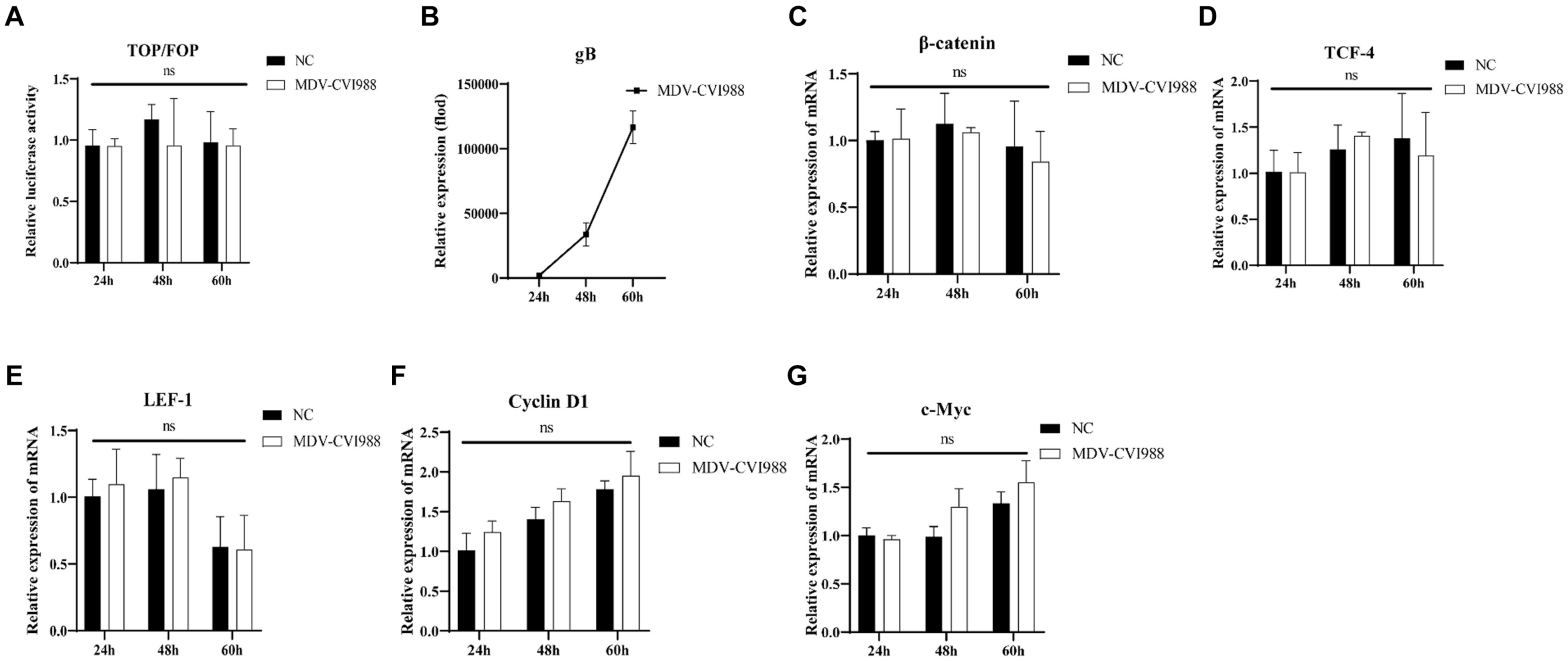
MDV CVI988 does not activate the Wnt/β-catenin Signaling Pathway in CEF. Relative activity of TOP/FOP luciferase in CEF cells at 24, 48, and 60 h post-inoculation with MDV-CVI988 strain **(A)**. Expression of MDV and Wnt/β-catenin signaling pathway-related genes in CEF cells at 24 h, 48 h, and 60 h post-inoculation with MDV-CVI988 strain detected by real-time RT-PCR **(B–G)**. Data were expressed as mean ± SD from three independent experiments and analyzed by Student’s *t*-tests. And each experiment was repeated three times (^**^p < 0.01, ^****^p < 0.0001).

After transfection of the dual-luciferase reporter plasmid in CEF for 12 h, the cells were infected with the MDV RB1B and CVI988 strains (MOI = 0.001) for 24, 48, and 60 h. The protein of the cells at different time point was collected, and a dual-luciferase reporter assay was conducted to measure transcriptional activity. The results showed that β-catenin/TCF transcriptional activity was significantly activated at 60 h post-infection, which indicating that the MDV RB1B strain could activate the pathway (*p* < 0.001) (Figure 1A). The RNA was extracted from infected CEF cells at the same time points for real-time PCR analysis. The results indicated that, with the replication of the MDV RB1B strain, the expression level of Wnt/β-catenin signaling pathway-related genes β-catenin, TCF4, LEF1, c-Myc, and Cyclin D1 were significantly downregulated after 24 h of RB1B strain infection and significantly upregulated at 60 h. This suggested that the pathway is initially inhibited and later activated following MDV RB1B infection (*p* < 0.05) (Figures 1B–G). However, CVI988 virus strain infection does not affect the Wnt/β-Catenin signaling pathway (*p*>0.05) (Figures 2A–G).

### MDV RB1B increases phosphorylation of GSK-3β in CEF cells

**Figure 3:**
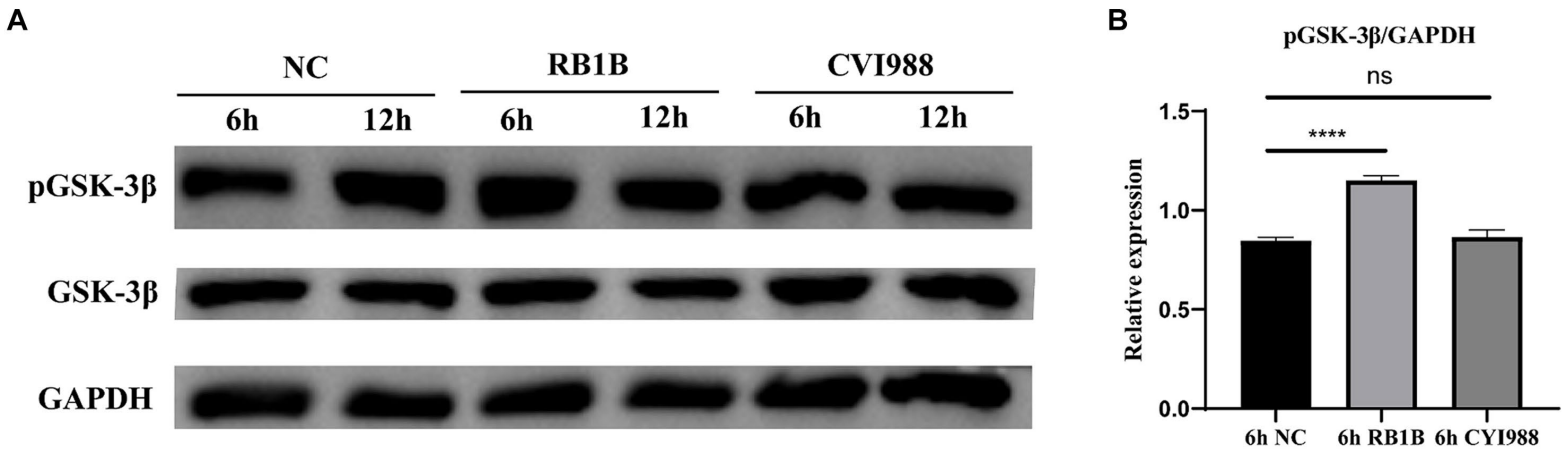
MDV RB1B increases phosphorylation of GSK-3β. CEF cells Were inoculated with RB1B and CVI988 Strains for 6 and 12 h, respectively. The protein expression levels of pGSK-3β and GSK-3β were measured by western blotting **(A)**. **(B)** Quantification of relative phosphorylated protein band intensities to GAPDH in **(A)**. The data represent the mean ± SD of three independent experiments and analyzed by Student’s *t*-tests. And each experiment was repeated three times (^****^p < 0.0001).

According to previous studies, GSK-3β was inactivated after phosphorylation of its ninth serine residue, thus activating the Wnt/β-catenin signaling pathway (Chen et al., 2019; Yang et al., 2022; Zhu et al., 2023). Therefore, we investigated the phosphorylation status of GSK-3β following infection of CEF cells with different strains of MDV. CEF cells were infected with MDV RB1B and MDV CVI988 strains (MOI = 0.001), and protein samples were collected at 6 and 12 h post-infection for Western Blot analysis. The results demonstrated that 6 h post-infection with the MDV RB1B strain increased in the phosphorylation level of GSK-3β, while MDV CVI988 had no effect. This suggests that MDV RB1B activates the Wnt/β-catenin signaling pathway by increasing the phosphorylation level of GSK-3β (*p* < 0.001) (Figure 3).

### *In vivo* activation of the Wnt/β-catenin signaling pathway by MDV RB1B infection, but not CVI988

**Figure 4:**
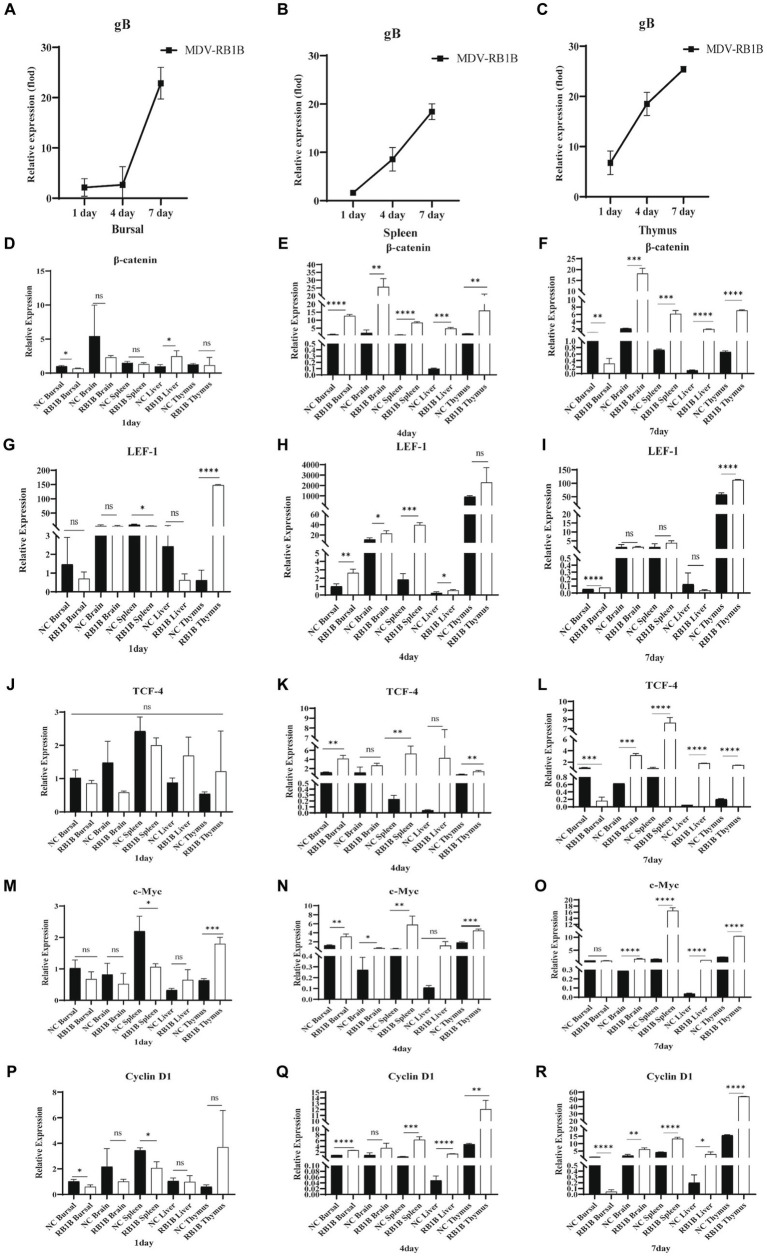
Infection with MDV RB1B activates the Wnt/β-catenin signaling pathway in SPF chicken. At 1, 4, and 7 days post-inoculation with RB1B strain, the expression levels of MDV **(A–C)** and Wnt/β-catenin signaling pathway-related genes in bursa of fabricius, brain, spleen, liver, and thymus were detected by real-time PCR **(D–R)**. Data were expressed as mean ± SD from three independent experiments and analyzed by Student’s *t*-tests. And each experiment was repeated three times (^*^p < 0.05, ^**^p < 0.01, ^***^p < 0.001, ^****^p < 0.0001).

**Figure 5:**
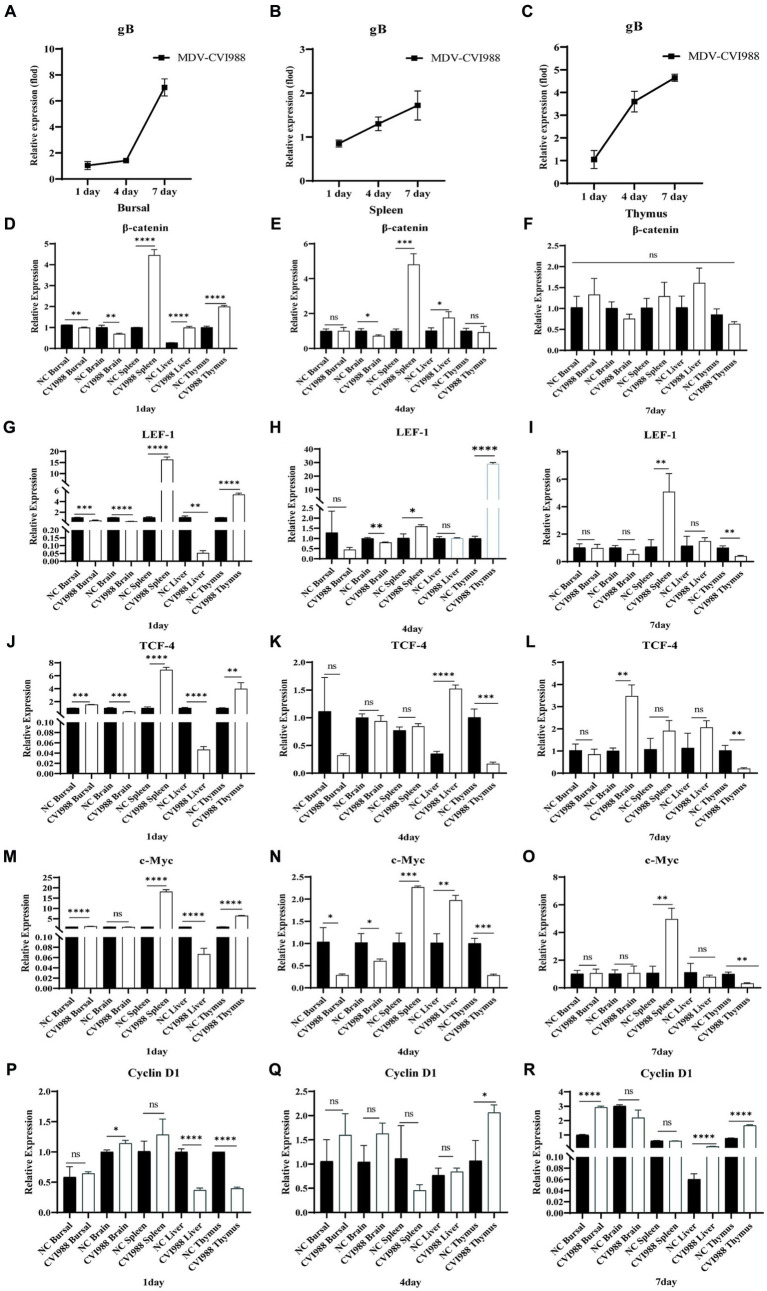
Infection with MDV CVI988 effects the Wnt/β-catenin signaling pathway in SPF chicken. At 1, 4, and 7 days post-inoculation with CVI988 strain, the expression levels of MDV **(A–C)** and Wnt/β-catenin signaling pathway-related genes in bursa of fabricius, brain, spleen, liver, and thymus were detected by real-time PCR **(D–R)**. Data were expressed as mean ± SD from three independent experiments and analyzed by Student’s *t*-tests. And each experiment was repeated three times (^*^p < 0.05, ^**^p < 0.01, ^***^p < 0.001, ^****^p < 0.0001).

To investigate the impact of MDV on Wnt/β-catenin signaling pathway *in vivo*, the expression of viral-encoded genes and signaling pathway-related genes were examined following SPF chickens infected with MDV RB1B and CVI988. One-day-old SPF chickens were inoculated with the MDV RB1B strain and CVI988 strain at a dose of 2000 PFU per-bird. Tissues, bursa of fabricius, spleen, brain, thymus, and liver, were collected for analysis at days 1, 4, and 7 post-infection. The tissues were collected from three chickens per-group at one time point. Tissue RNA was extracted and reverse-transcribed. The expression of viral-encoded genes and Wnt/β-catenin pathway-related genes were detected by real-time PCR. The results showed that as the viral infection progressed, the expression of Wnt/β-catenin pathway-related genes β-catenin, TCF-4, LEF-1, c-Myc, and Cyclin D1 were significantly upregulated in RB1B infection group (Figure 4). However, in SPF chickens, CVI988 had a weaker influence on the Wnt/β-catenin signaling pathway compared to RB1B, exhibiting a different trend, possibly related to the protective effect induced by the vaccine strain (Figure 5).

### Impact of Meq proteins encoded by different MDV strains on the Wnt/β-catenin signaling pathway

**Figure 6:**
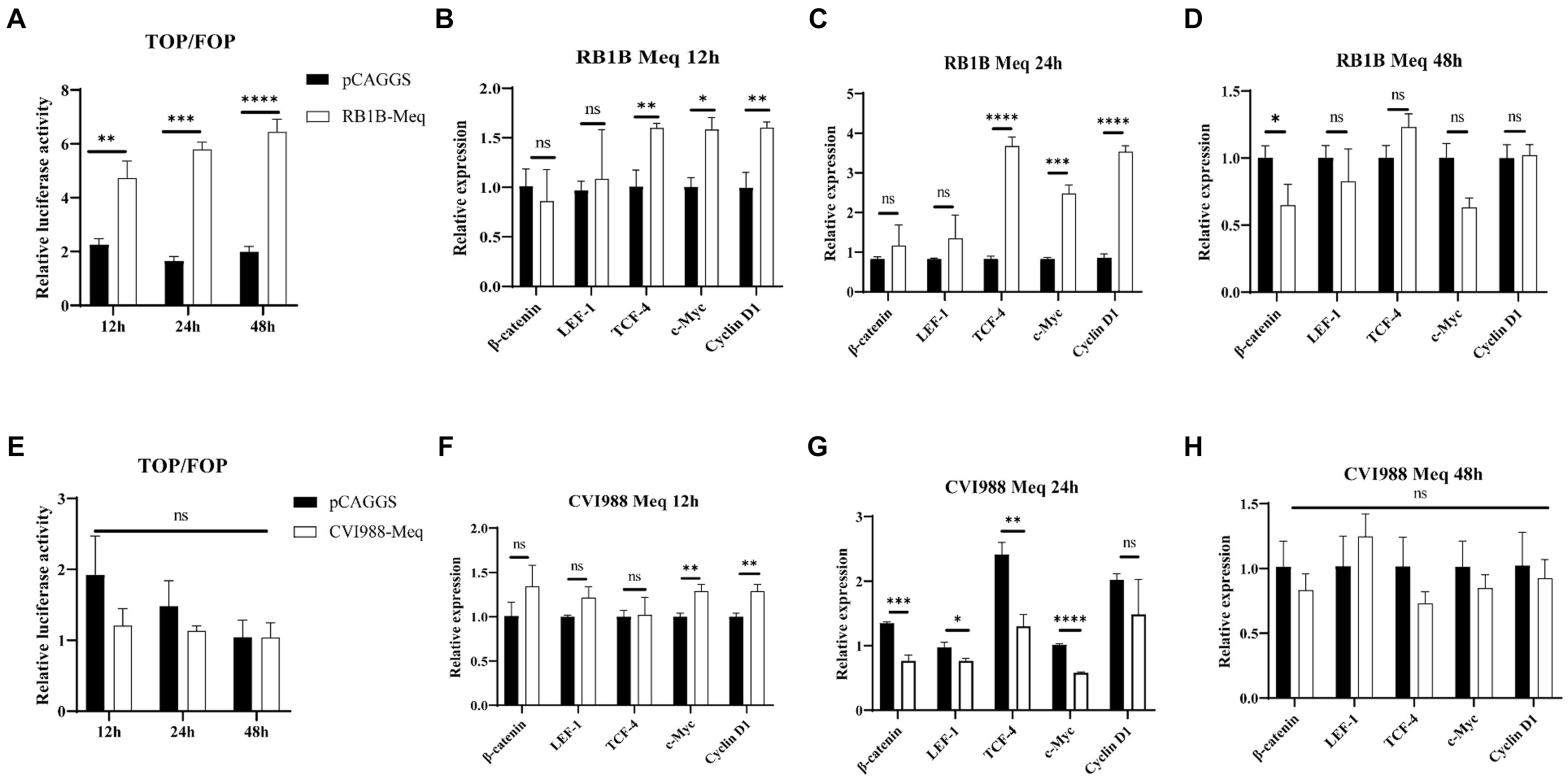
The effect of Meq protein of different MDV strains on the Wnt/β-catenin Signaling Pathway. After transfection with pCAGGS-RB1B-Meq in CEF for 12, 24, and 48 h, the relative activity of TOP/FOP luciferase was measured **(A)**. The expression levels of Wnt/β-catenin signaling pathway-related genes were detected by real-time PCR at 12, 24, and 48 h after pCAGGS-RB1B-Meq transfection **(B–D)**. Following transfection with pCAGGS-CVI988-Meq in CEF for 12, 24, and 48 h, the relative activity of TOP/FOP luciferase was detected **(E)**. The expression levels of Wnt/β-catenin signaling pathway-related genes were measured by real-time PCR at 12, 24, and 48 h after pCAGGS-CVI988-Meq transfection **(F–H)**. Data were expressed as mean ± SD from three independent experiments and analyzed by Student’s *t*-tests. And each experiment was repeated three times (^*^p < 0.05, ^**^p < 0.01, ^***^p < 0.001, ^****^p < 0.0001).

The Meq protein encoded by the MDV virus is closely associated with tumor induction upon viral infection (Conradie et al., 2020). In this study, we selected the Meq protein to investigate the influence of MDV-encoded proteins on the Wnt/β-catenin signaling pathway. After transfecting CEF cells with pCAGGS-RB1B-Meq plasmid for 12, 24, and 48 h, cell proteins were collected and a dual-luciferase assay was performed. The results showed that Meq protein from RB1B strain strongly promoted the binding of β-catenin to the TCF/LEF promoter in CEF cells (Figure 6A). Cell RNA was collected and reverse-transcribed for real-time PCR to detect the expression of Wnt/β-catenin pathway-related genes. The results showed that Meq from RB1B strain upregulated the expression of Wnt/β-catenin pathway-related genes at 12 and 24 h post-transfection (Figures 6A–D). In contrast to the Meq protein encoded by RB1B, the Meq protein from CVI988 not only had no effect on the β-catenin/TCF transcriptional activity (Figure 6E), but also inhibited the expression of pathway-related genes at 24 h post-transfection (Figures 6F–H). These results indicate that in CEF cells, Meq protein encoded by MDV RB1B can significantly activate the Wnt/β-catenin signaling pathway.

### iCRT14 inhibits the activation of Wnt/β-catenin signaling pathway by Meq protein from MDV RB1B

**Figure 7:**
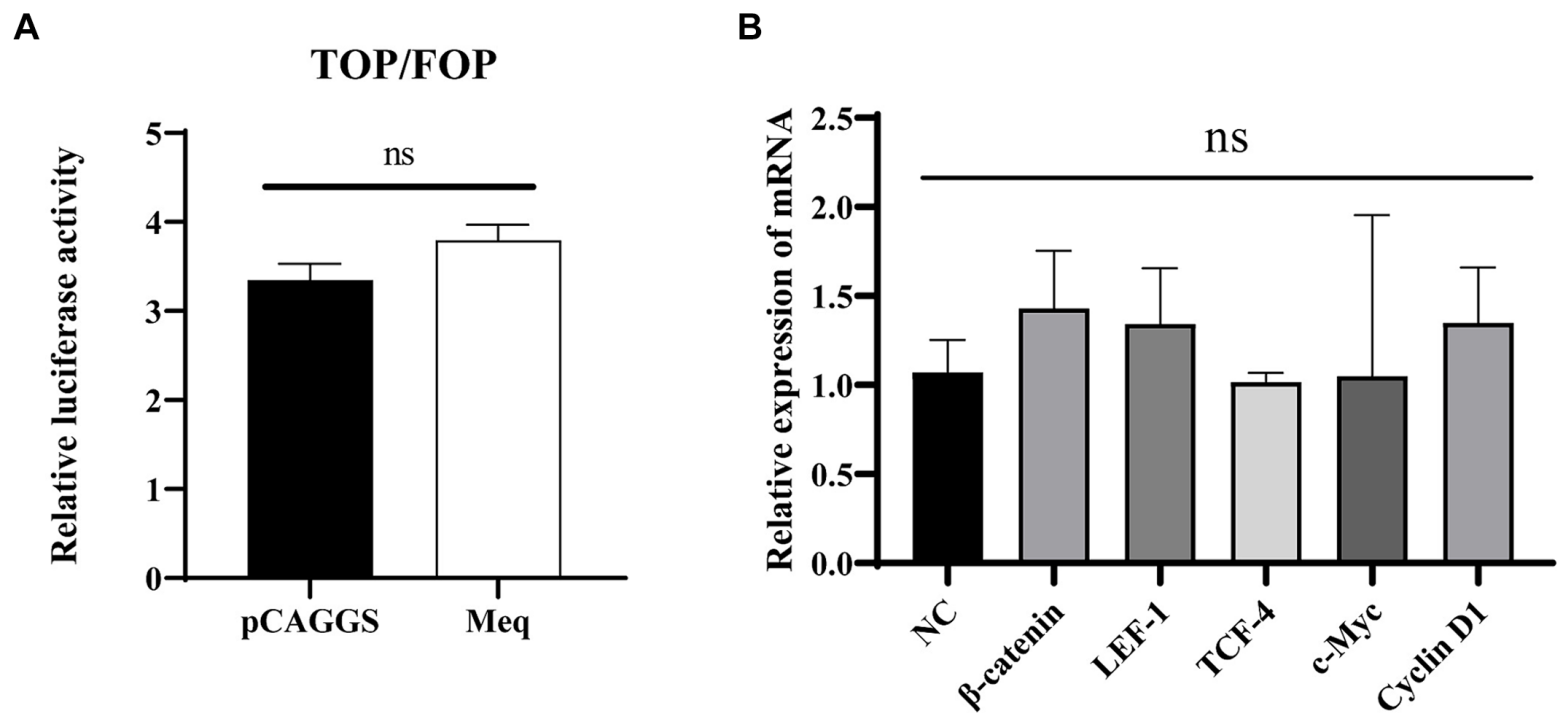
iCRT14 inhibits the activation of the Wnt/β-catenin signaling pathway by Meq protein of MDV RB1B strain. After transfecting CEF with pCAGGS-RB1B-Meq for 12 h, cells were treated with iCRT14, and the relative activity of TOP/FOP luciferase was measured 24 h post-transfection **(A)**. Following the transfection of CEF with pCAGGS-RB1B-Meq for 12 h and treatment with iCRT14, the expression levels of Wnt/β-catenin signaling pathway-related genes were detected by real-time PCR at 24 h post-transfection **(B)**. Data were expressed as mean ± SD from three independent experiments and analyzed by Student’s *t*-tests. And each experiment was repeated three times.

According to the previous report (Qiao et al., 2021), we chose iCRT14, a Wnt signaling inhibitor that disrupts the interaction between β-catenin and TCF4, to further confirm the specific activation of the Wnt/β-catenin signaling pathway by the Meq protein. The cells were treated with iCRT14 following 12 h post-transfection of the plasmid, and samples were collected at 24 h post-transfection for real-time PCR and dual-luciferase reporter assay. The results indicated that after treatment with the pathway inhibitor, Meq protein had no effect on the expression of pathway-related genes. Consistently, the dual-luciferase reporter assay results showed no activation also, which demonstrating that the pathway inhibitor iCRT14 can inhibit the activation of the Wnt/β-catenin signaling pathway by Meq encoded by MDV RB1B (*p* > 0.05) (Figure 7). This suggests that Meq protein encoded by MDV RB1B can specifically activate the Wnt/β-catenin signaling pathway.

## Discussion

The MDV infection leads to immunosuppression and induces tumor formation. There have been some reports on the mechanisms of virus-induced tumorigenesis, but the understanding is not comprehensive yet (Burgess et al., 2004; Baigent et al., 2011, 2016). The results of this study supplement our understanding of the regulation of Wnt/β-catenin signaling by MDV. *In vitro* cellular infection experiments showed that MDV RB1B infection activated the Wnt/β-catenin signaling pathway in cells, while MDV CVI988 infection did not. Further studies revealed that the RB1B strain activated the Wnt/β-catenin pathway by increasing the phosphorylation level of in GSK-3β, resulting in the functional inactivation of GSK-3β and thus activating the Wnt/β-catenin signaling pathway. However, the vaccine strain CVI988 did not exhibit similar effects. GSK-3β, as a multifunctional protein in the host, plays an important role in various signaling pathways. The phosphorylation of GSK-3β is considered a hallmark of the activation of the Wnt/β-catenin signaling pathway, as has been reported in numerous articles (Chen et al., 2019; Yang et al., 2022; Zhu et al., 2023). The impact of MDV on other biological function changed through GSK-3β phosphorylation remains to be further investigated. In the subsequent *in vivo* experiments, the results showed that under normal conditions of virus infection and replication, the MDV RB1B strain overall upregulated genes related to the Wnt/β-catenin signaling pathway, while the MDV CVI988 strain exhibited a general downward trend, with significant gene expression inhibition observed in some tissues. Although the expression of some genes in the CVI88 infection group increased at the initial stage of infection, they all showed a downward trend, which was completely different from that in the RB1B group. Combining the above experimental results indicates that MDV RB1B is capable of activating the Wnt/β-catenin signaling pathway, whereas MDV CVI988 does not.

Previous studies have indicated that many viruses influence the Wnt signaling pathway through encoded viral proteins. The VP1 protein encoded by Porcine Circovirus could inhibit the expression of pathway target genes by preventing β-catenin nuclear entry and deactivating the TCF/LEF promoter activity (Zhu et al., 2018). The HBV core protein P22 could activate the Wnt/β-catenin signaling pathway in a cancerous environment (Tran et al., 2020). The core protein and non-structural protein 4B of Hepatitis C Virus (HCV) induced β-catenin nuclear entry, thereby activating the Wnt/β-catenin signaling pathway (Jiang et al., 2017). Based on the influence of MDV virus on the Wnt/β-catenin signaling pathway both *in vitro* and *in vivo*, we further investigated whether the MDV virus’s main oncogenic gene, Meq, would affect the activity of the signaling pathway. The Meq proteins encoded by the RB1B and CVI988 strains of MDV indeed had different effects on the Wnt/β-catenin signaling pathway. The Meq protein from the RB1B strain could activate the Wnt signaling pathway in cells, while the Meq protein from the CVI988 strain could not. Sequence comparison between the RB1B and CVI988 strains encoding Meq revealed a 178 bp insertion sequence in the Meq of the CVI988 strain (Subramaniam et al., 2013). Whether this difference of 178 bp insertion is related to the influence on the Wnt signaling pathway of Meq protein and the role of other MDV-encoded proteins in the Wnt/β-catenin signaling pathway requires further research to confirm.

In conclusion, this study verified the impact of MDV on the Wnt/β-catenin signaling pathway for the first time *in vivo* and *in vitro*. Furthermore, it was discovered that the Meq protein encoded by the RB1B strain played a role in activating the signaling pathway. This research provided new insights into the virus–host interactions of MDV.

## Data availability statement

The original contributions presented in the study are included in the article/supplementary material, further inquiries can be directed to the corresponding authors.

## Ethics statement

The animal study was approved by Animal Care Committee of Yangzhou University in China. The study was conducted in accordance with the local legislation and institutional requirements.

## Author contributions

HX: Data curation, Formal analysis, Methodology, Software, Writing – original draft. XX: Data curation, Formal analysis, Methodology, Software, Writing – review & editing. HH: Formal analysis, Methodology, Software, Writing – original draft. HS: Writing – review & editing. YY: Funding acquisition, Writing – review & editing. AQ: Funding acquisition, Writing – review & editing. KQ: Data curation, Formal analysis, Funding acquisition, Investigation, Project administration, Supervision, Writing – review & editing.
